# Supplementary figures and images for: Immune status changing helps diagnose osteoarticular tuberculosis
Source: PLoS One. 2021 Jun 15;16(6):e0252875. doi: 10.1371/journal.pone.0252875 (PMC8205131; doi:10.1371/journal.pone.0252875)

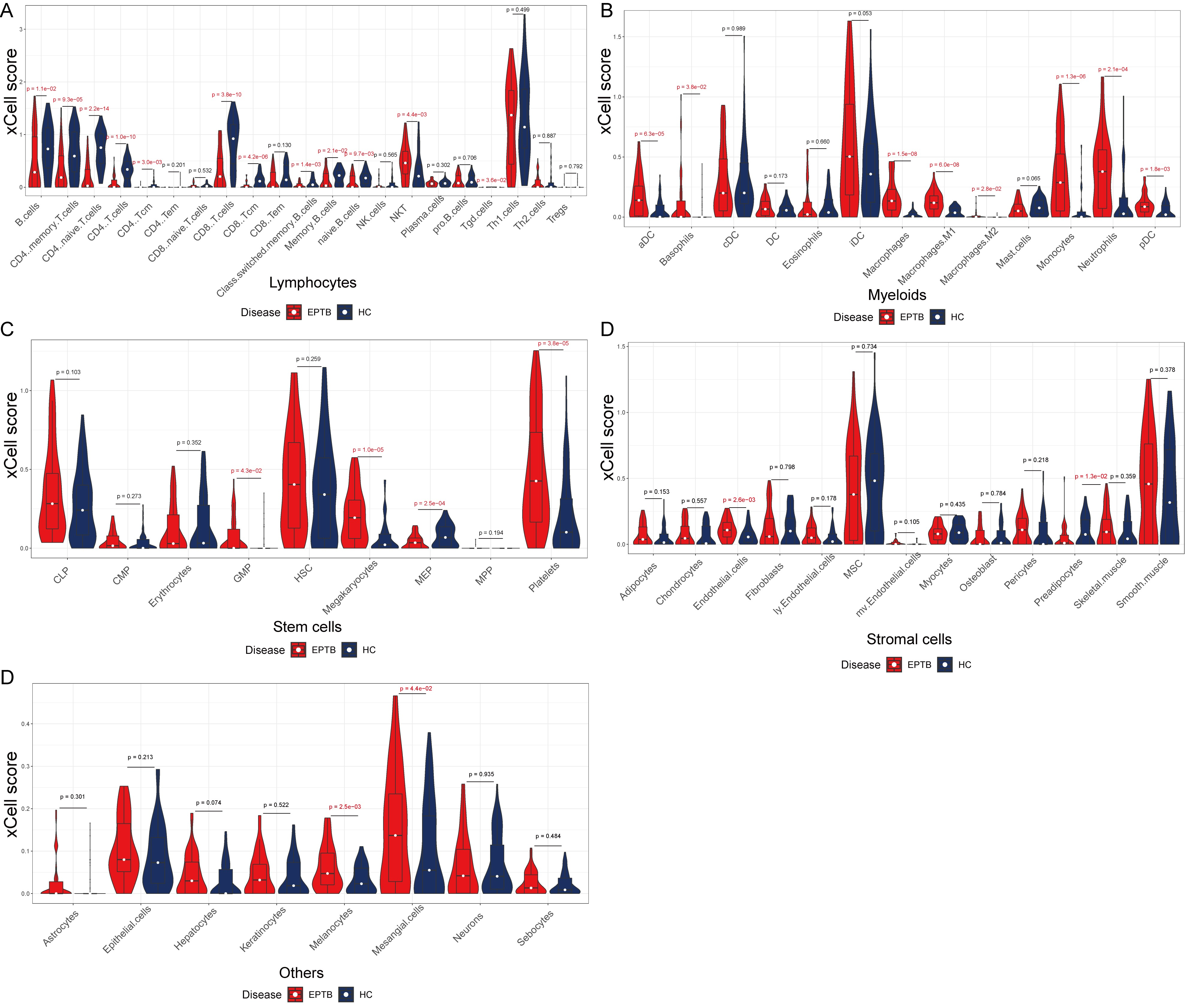

Supplement: S1 Fig — (A, B, C, D, E) xCell score of sixty-four cell types in GSE83456 were grouped into five groups: lymphocytes, myeloids, stem, stromal, and other cells. (TIF) [file pone.0252875.s001.tif]

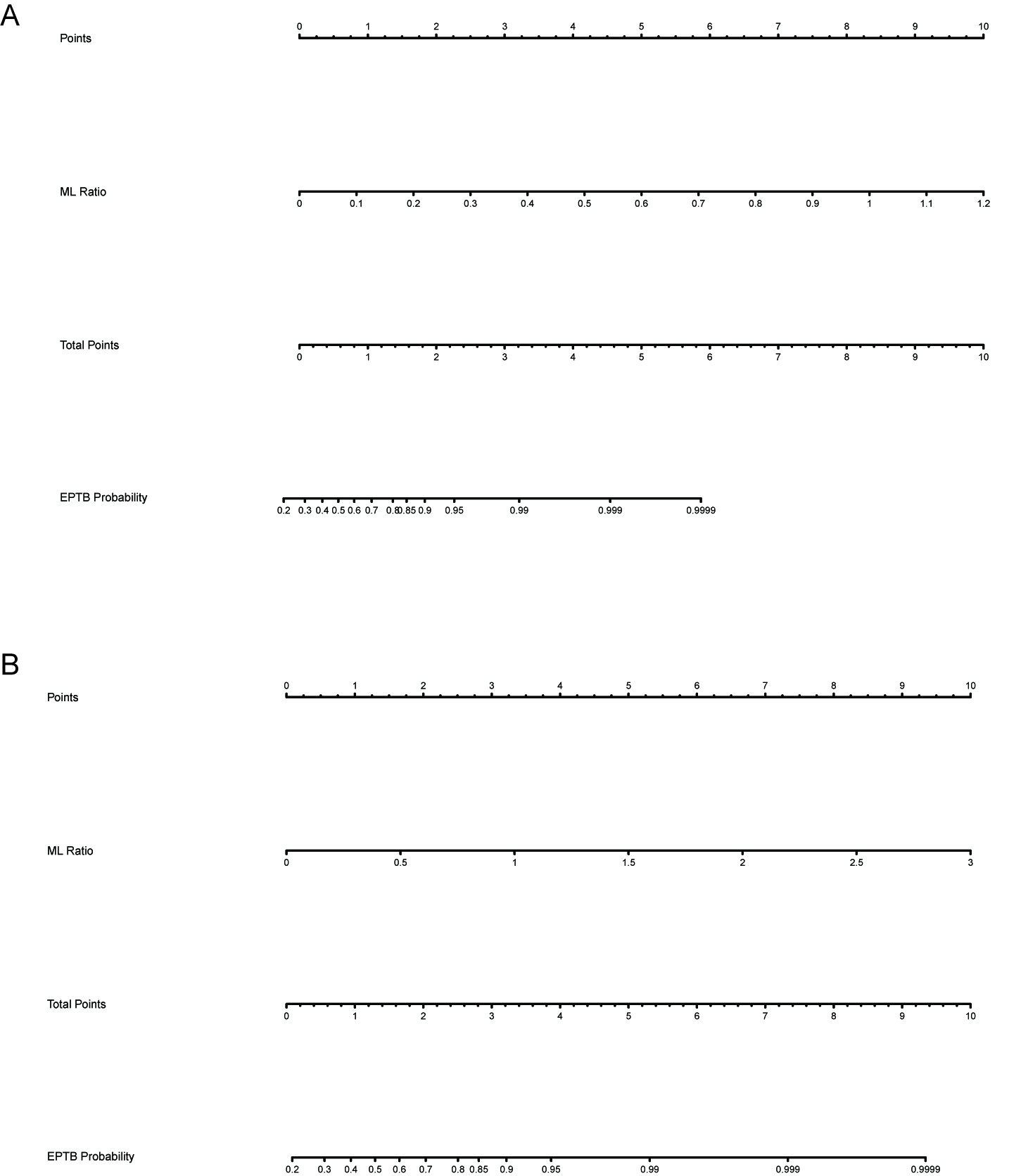

Supplement: S2 Fig — (A) Nomogram for predicting EPTB probability base on ML ratio in dataset GSE83456. (B) Nomogram for predicting osteoarticular TB probability base on ML ratio in clinical data. (TIF) [file pone.0252875.s002.tif]
