# Supplementary material for: Immune status changing helps diagnose osteoarticular tuberculosis
Source: PLoS One. 2021 Jun 15;16(6):e0252875. doi: 10.1371/journal.pone.0252875 (PMC8205131; doi:10.1371/journal.pone.0252875)
Supplement: Attachment — Submitted filename: revision letter.docx [file pone.0252875.s004.docx]

Dear Editors and Reviewers:

It is with excitement that I resubmit to you a revised version of the manuscript “**Immune Status Changing Helps Diagnose Osteoarticular Tuberculosis**” (ID: PONE-D-20-39926) for the “**Plos One**”. Thank you for giving me the opportunity to revise and resubmit this manuscript. I appreciate the time and detail provided by each reviewer and by you and have incorporated the suggested changes into the manuscript to the best of my ability. We highlight the changes to our manuscript within the document by using the track changes mode in Microsoft Word. The manuscript has certainly benefited from these insightful revision suggestions. I look forward to working with you and the reviewers to move this manuscript closer to publication in the “**Plos One**”. According to your nice suggestions, we have made extensive corrections to our previous draft. changes to the manuscript are shown in red. Point-by-point responses to the nice associate editor and three nice reviewers are listed below this letter.

Responses to the reviewer’s comments:

**Reviewer #1:**

**Major points**

*1.* *About the enrolled patients, it is not clear how the diagnosis of bone tuberculosis was made. The authors, in the material and methods section and table 1, should provide data relating to immunological tests, even if partial, or in any case, clarify how the diagnosis of bone tuberculosis was reached, e.g. by imaging.*

**Thank you for your valuable comments. We redefined the diagnostic and exclusion criteria for osteoarticular TB (Methods section, line 93-103, page 4).**

*2. In lines 27, 57, 71, etc. the authors refer to lymphoid cells and not lymphocytes, in supplementary table 1 there is no a lymphocyte population as a whole but 21 different populations classified as lymphoid; the authors should specify which of these populations were used for the calculation of the ML ratio obtained from the GSE83456 dataset and which differences, in terms of calculation, exist between this ML ratio and that obtained from the clinical data of the patients.*

**Thank you for your valuable comments. Consistenting with the method of lymphocyte count in the routine blood examination, the xCell score of lymphocytes was a constitution of 21** **subtypes (Supplemental Figure S1) of lymphoid cells (B cells, T cells and NK cells). We also point out that in the main text (Methods section, line 83-86, page 3). The ML ratio in the dataset GSE83456 represents the ratio of xCell score of monocytes to lymphocytes. The ML ratio in the clinical dataset represents the ratio of cell counts of monocytes to lymphocytes (Results section, line 152-154, page 6).**

*3. In Figure 2A it is not clear to me how the ML ratio was calculated from the GSE83456 dataset, is it a ratio between the scores?*

**Thank you for your valuable comments. The ML ratio in the dataset GSE83456 represents the ratio of xCell score of monocytes to lymphocytes. The ML ratio in the clinical dataset represents the ratio of cell counts of monocytes to lymphocytes (Results section, line 152-154, page 6).**

*4. In lines 105 and 106, the authors refer to healthy controls who actually are subjects suffering from disc herniation or lumbar spinal stenosis, for clarity it would be better to define them as non-TB patients.*

**Thank you for your valuable comments. In The First Affiliated Hospital of Guangxi Medical University, from 2012 and 2018, we randomly screened out the non-TB patients from all the inpatients diagnosed with lumbar disc herniation or lumbar spinal stenosis (Methods section, line 105-107, page 4).**

*5. In figure 1C the authors refer to a cell ratio, but it is not clear what this parameter indicates, is it perhaps the frequency of the single populations on the total of leukocytes?*

**Thank you for your valuable comments. The cell ratio represents the frequency of the single populations on the total of leukocytes in the routine blood examination. Actually, these data had no effect on the results in this study. We have deleted figure 1C from figure 1 in this version of manuscript.**

*6. In figure S1 there are two nomograms (A and B) but in the text, they are not described and it is not clear what difference there is between the two; the authors should clarify this point.*

**Thank you for your valuable comments. We identified the ML ratio as an independent risk factor for the diagnosis of EPTB; therefore, nomograms (Supplemental Figure S2) based on the ML ratio were constructed for both dataset GSE83456 and clinical data (Results section, line 155-159, page 6). Figure S2A represents the nomogram for predicting EPTB probability base on ML ratio in dataset GSE83456. Figure S2B represents the nomogram for predicting osteoarticular TB probability base on ML ratio in clinical data (Figure legends, line 442-444, page 16).**

**Minor points**

*1. In table 1, please correct the term eosinophil, in general table 1 should be better formatted.*

**Thank you for your valuable comments. We have modified this part in the text (Table 1).**

*2. Would be interesting if the authors, in fig 3A show where some NPTB and non TB patient lies in the nomogram.*

**Thank you for your valuable comments. We have modified this part in the text (Figure 3A). The red line represents an osteoarticular TB patient, while the green line represents a non-TB patient.**

*3.* *The right citation for reference n15 is: La Manna MP, Orlando V, Dieli F, Di Carlo P, Cascio A, Cuzzi G, Palmieri F, Goletti D, Caccamo N. Quantitative and qualitative profiles of circulating monocytes may help identifying tuberculosis infection and disease stages. PLoS One. 2017 Feb 16;12(2):e0171358. doi: 10.1371/journal.pone.0171358. Among the authors, there is no Wilkinson KA.*

**Thank you for your valuable comments. We have modified this part in the text as reference no.13 (Reference section, line 337-341, page 12).**

**Reviewer #2**

*Need to be re written. This manuscript does not reflect new findings. In addition, there a wrong claim in it. The authors said that bone infection is the most common extrapulmonary Tb. This statement contradicts most of the publications which said lymph node is the most dominant extrapulmonary type. The English language is very poor.*

**Thank you for your valuable comments. Due to the lack of writing and English ability, the article before was poorly organized, so we search professional help for** **improvements to the English language within our manuscript.**

**Reviewer #3**

*1.The manuscript requires a lot of English editing, and some sections need revision.* *There are many ambiguities in method, result and discussion sections which need careful review and revision, given below are few examples.*

*Method: Type of demographic, clinical and laboratory data collected from TB and non-TB patient files (2012-2018) and source of cell counts used for comparison with GSE83456 data file.*

*Results: Vague expressions and unspecific determinate words ["much lower”, “much higher” “satisfactory agreement”] are used that are not specific or precise enough for the reader to derive exact meaning.*

**Thank you for your valuable comments. Due to the lack of writing and English ability, the article before was poorly organized, so we search professional help for** **improvements to the English language within our manuscript.**

**Minor comments**

*1.* *For some statements, references are not cited (row 50-52 and 214-215)*

**Thank you for your valuable comments. We have modified this part in the text.**

*2.* *Tables; Abbreviation used and unit of measurements are not described.*

**Thank you for your valuable comments. We have modified this part in the text (Table 1).**

We tried our best to improve the manuscript and made some changes in the manuscript. These changes will not influence the content and framework of the paper. And here we did not list the changes but marked in red in revised paper. We appreciate for Editors/Reviewers’ warm work earnestly, and hope that the correction will meet with approval.

Once again, thank you very much for your comments and suggestions.
